# Achievements and Challenges in Transplantation of Mesenchymal Stem Cells in Otorhinolaryngology

**DOI:** 10.3390/jcm10132940

**Published:** 2021-06-30

**Authors:** Reza Kaboodkhani, Davood Mehrabani, Feridoun Karimi-Busheri

**Affiliations:** 1 Otorhinolaryngology Research Center, Department of Otorhinolaryngology, School of Medicine, Shiraz University of Medical Sciences, Shiraz 71936-36981, Iran; kaboodkhani@gmail.com; 2 Stem Cell Technology Research Center, Shiraz University of Medical Sciences, Shiraz 71348-14336, Iran; 3 Burn and Wound Healing Research Center, Shiraz University of Medical Sciences, Shiraz 71987-74731, Iran; 4 Comparative and Experimental Medicine Center, Shiraz University of Medical Sciences, Shiraz 71348-14336, Iran; 5 Li Ka Shing Center for Health Research and Innovation, University of Alberta, Edmonton, AB T6G 2E1, Canada; 6 Department of Oncology, Faculty of Medicine, University of Alberta, Edmonton, AB T6G 1Z2, Canada

**Keywords:** mesenchymal stem cells, transplantation, otorhinolaryngology, head and neck surgery

## Abstract

Otorhinolaryngology enrolls head and neck surgery in various tissues such as ear, nose, and throat (ENT) that govern different activities such as hearing, breathing, smelling, production of vocal sounds, the balance, deglutition, facial animation, air filtration and humidification, and articulation during speech, while absence of these functions can lead to high morbidity and even mortality. Conventional therapies for head and neck damaged tissues include grafts, transplants, and artificial materials, but grafts have limited availability and cause morbidity in the donor site. To improve these limitations, regenerative medicine, as a novel and rapidly growing field, has opened a new therapeutic window in otorhinolaryngology by using cell transplantation to target the healing and replacement of injured tissues. There is a high risk of rejection and tumor formation for transplantation of embryonic stem cells (ESCs) and induced pluripotent stem cells (iPSCs); mesenchymal stem cells (MSCs) lack these drawbacks. They have easy expansion and antiapoptotic properties with a wide range of healing and aesthetic functions that make them a novel candidate in otorhinolaryngology for craniofacial defects and diseases and hold immense promise for bone tissue healing; even the tissue sources and types of MSCs, the method of cell introduction and their preparation quality can influence the final outcome in the injured tissue. In this review, we demonstrated the anti-inflammatory and immunomodulatory properties of MSCs, from different sources, to be safely used for cell-based therapies in otorhinolaryngology, while their achievements and challenges have been described too.

## 1. Introduction

Head and neck structures are responsible for vital activities of swallowing and breathing and facilitate our sense of self by vocal communication, physical appearance, facial animation, and hearing, while lack of these activities can influence the quality of life and result in loss of life. In head and neck diseases and disorders, patients are expected to refer to an otorhinolaryngologist to search for treatment of damaged tissues in head and neck structures because the otorhinolaryngology field enrolls head and neck surgery in various tissues such as ear, nose and throat (ENT) that govern different activities such as hearing, breathing, smelling, production of vocal sounds, the balance, deglutition, facial animation, air filtration and humidification, and articulation during speech. Therefore, absence of these functions can lead to high morbidity and even mortality [1,2].

Conventional therapies for head and neck damaged tissues include artificial materials and grafts from other tissues [2], but grafts were shown to have limited availability and can lead to morbidity in the donor site [3], and the use of artificial materials can have the risk of infection and reaction by the immune system [1]. When grafts are undertaken, immunosuppressive drugs are needed that have limited availability in many regions [4]. To improve these limitations, regenerative medicine, by using cell transplantation, has opened a new therapeutic window, which is a novel and rapidly growing field in otorhinolaryngology, which targets the healing and replacement of injured tissues where no current standard therapy works to restore functions of otorhinolaryngology sites [5]. In this review, we described achievements and challenges in regenerative medicine research using cell transplantation in otorhinolaryngology and head and neck surgery fields.

## 2. Sources and Selection Criteria

Articles published in PubMed and Scholar Google from 2003 to 2021 were searched using search terms: “stem cell”, “cell transplantation”, “regenerative medicine”, “scaffold” and “tissue engineering” with “ear”, “hearing”, “tympanic membrane”, “cochlea”, “nose”, “vocal fold”, “larynx”, “sinus”, “craniofacial”, and “head and neck”.

## 3. Regenerative Medicine and Cell Transplantation Concept

Regenerative medicine covers “the process of replacing, engineering or regenerating human cells, tissues or organs to restore the tissue or organ normal function” [6]. In regenerative medicine, in vivo regeneration of tissues happens by use of human body as a bioreactor to augment the body’s innate ability to regenerate and heal. In the otorhinolaryngology field, regenerative medicine utilizes cell transplantation and scaffolds based on tissue type and activity in head and neck regions. A wide range of variations exist. Scaffolds can provide a three-dimensional structure to induce cell migration and differentiation to restore normal organ function [7]. Scaffolds can be prepared by using a wide range of methods, such as decellularizing tissue [8], three-dimensional printing [9], customizing hydrogels [10], and electrospinning [11]. In otorhinolaryngology, biocompatible scaffolds have been used to establish a normal structure and function in an injured tissue [12]. Cell transplantation or cell therapy is the other branch utilized in regenerative medicine to modulate immune response and regenerate new tissues, via paracrine signaling, by use of various types of stem cells, including embryonic stem cells (ESCs), induced pluripotent stem cells (iPSCs), and mesenchymal stem cells (MSCs) [4]. In cell-based therapies, replacement of the lost or damaged tissues happens via engraftment of viable transplanted cells and stimulation of endogenous self-healing pathways by trophic factors [13,14].

## 4. The Characteristics of MSCs

MSCs have opened a new window in regenerative medicine based on their easy expansion and wide range of healing and aesthetic functions [15]. They are non-hematopoietic cells that are plastic-adherent and spindle shape with self-renewing, migration, and differentiation properties [16]. They express mesenchymal surface markers such as CD44, CD73, CD90, and CD105, but they lack expression of hematopoietic markers such as CD34 and CD45 [17]. Various sources were mentioned for the isolation of MSCs including adipose tissue [18], bone marrow [19], and dental pulp [20]. The tissue sources and types of MSCs (autologous or allogeneic), the method of cell introduction (dosage, route, schedule, etc.), and their preparation quality can influence the final outcome in the injured tissue [21]. MSCs can change host immune responses via secretion of immune-modulatory proteins, limit inflammation through cytokine release, home to the site of injury, secrete anti-apoptotic factors, and stimulate healing in the injured tissue [22]. There are still challenges in in vitro expansion of stem cells that are needed to be overcome, such as the time-consuming nature of cell culture that can decrease the cell differentiation potential, due to epigenetic changes that may happen in cultured cells [23]. The donor site for cell isolation can affect the proliferation and differentiation potential of the isolated MSCs [24].

The advantage of MSCs is their anti-inflammatory characteristic that can be explained by the secretion of potent immunoregulatory factors preventing the proliferation and activity of T-helper 1 (Th1)/Th17 cells improving the Treg differentiation and leading to an increase in secretion of anti-inflammatory cytokines such as interleukin-4 (IL-4), IL-10, IL-11, IL-13, and transforming growth factor beta (TGF-β), and a decrease in inflammatory cytokines such as IL-6, IL-12, IL-21, IL-23, and nuclear factor kappa-light-chain-enhancer of activated B cells (NF-κB) activities. MSCs can directly inhibit the antigen-presenting function of dendritic cells and macrophages [25].

## 5. Cell Transplantation Advances in Otorhinolaryngology

Across otorhinolaryngology and head and neck surgery fields, advances in cell transplantation research and therapies have been different. Here we have discussed the achievements and challenges in the field of otorhinolaryngology and head and neck surgery, separately for each organ.

## 6. External Ear and the Auricle

The auricle or auricula or pinna is the visible part of the ear located outside the head. It is an avascular tissue and a surface organ of the human body with great importance in facial aesthetics. So, when the ear becomes damaged and deformed, there is a strong desire for reconstruction of the auricle, even it has limited self-repair and regenerative ability because of it lacking blood vessels and nerves [26]. Current treatments to repair auricular cartilage defects are the transplantation of autologous cartilage from ribs and the use of artificial prosthesis or implants, but there are some limitations in use of autologous costal cartilage such as tissue calcification, contracture, deformation, and absorbance over time [27].

Also, transplantation of autologous cartilage not only poses considerable trauma and complications, but it also needs very skilled surgeons to cover the aesthetic demands of patients [28]. Fortunately, the introduction of regenerative medicine, by using cell transplantation and tissue engineering, has offered an alternative strategy to overcome these obstacles and produce fibrocartilaginous tissue that could satisfy the needs for structural support and graft durability and to repair or replace the damaged cartilage. Bahrani et al. reported successful differentiation of adipose-derived stem cells (AdSCs) into ear auricle cartilage in rabbits [29]. Auricular cartilage tissue engineering dates back to the year 2000 using polyglycolic acid (PGA) scaffolds seeded with chondrocytes for formation of a neocartilage tissue resembling the histological features of a natural cartilage [30].

There is paucity in the literature regarding auricular reconstruction including cases of microtia, especially characterizing historical mistakes, significant technical evolutions, and challenges of materials and chondrocytes. Microtia is an underdeveloped ear and a rare congenital dysmorphology affecting the development of the outer ear; while different degrees of reduction in size and malformed shape are visible among microtic ears based on the severity of the malformation [31]. Therefore, as chondrocytes have limited proliferation, low metabolic activity and the harvesting of the cartilage source causes the risk of complications, MSCs have been added to chondrocytes to yield enough cartilage constructs for tissue engineering, such as pressed PGA fiber mesh, coated with poly-lactic acid (PLA) or collagen gel scaffolds [32,33].

Chondrogenic differentiation property of MSCs needs a special medium containing several cytokines, such as TGF-β1, TGF- β2, TGF-β3, bone morphogenetic protein (BMP)-2, BMP-6, BMP-7, insulin-like growth factor 1 (IGF-1), platelet-derived growth factor subunit b (Pdgf-b), and fibroblast growth factor-2 (FGF-2) [34,35,36]. SRY-Box transcription factor 9 (Sox-9) is also a key regulator of MSCs in chondrogenesis and generation of spherical immature chondrocytes containing primordial cartilage [37]. For cartilage defects, cartilage stem/progenitor cells (CSPCs) have been successfully used for reconstructive purposes and for modeling the etiopathogenesis of microtia [31,38]. Ogawa et al. demonstrated chondrogenic differentiation of AdSCs in a three-dimensional collagen scaffold [39].

## 7. Inner Ear and Hearing Loss

It is estimated that 15% of the world’s population, of which over 90% are adults, suffer from some degree of hearing loss [40], and 10–14% of the world’s population are expected to develop hearing loss during their lifetimes [41]. According to the World Health Organization’s (WHO) estimate [40], the prevalence of hearing impairment is expected to increase from 460 million individuals in 2019 to more than 900 million people by 2050. Hearing loss is the loss of function in the inner ear that happens due to environmental exposures, acoustic overexposures, ototoxic medications such as cisplatin and aminoglycoside, and gene mutations responsible for hearing and aging [14].

The inner ear is composed of the cochlea and vestibular organs (saccule, utricle, and three semicircular canals), while each part harbors mechanosensory hair cells (HCs) that convert the mechanical stimuli (i.e., head motion or sound) into electrical signals in the afferent sensory neurons via synaptic transmission. The electrical signals in the vestibulocochlear nerve are further transmitted to the brain stem and then to the auditory cortex in the brain. An injury to the vestibulocochlear nerve, the sensory HCs, or the synaptic connections between them can lead to hearing loss and/or vertigo [14].

In hearing loss, HCs and spiral ganglion neurons (SGNs) are usually damaged and divide hearing loss into conductive and sensorineural types. In conductive hearing loss, which is a biophysical problem, fixation or disruption of the ossicular chain, middle ear effusion, and third window of the cochlea happens but are usually surgically treated. In sensorineural hearing loss (SNHL), which is the most common form and accounts for 90% of all hearing loss diagnoses, patients may experience tinnitus as the most prominent symptom. In SHNL, the loss of sensory HCs or damage involving the afferent nerve pathway to the auditory cortex occurs and is treated with hearing devices, ranging from externally worn to implantable devices that are mostly irreversible, and results in a permanent hearing loss [42,43]. The most common treatment choice of SNHL is hearing rehabilitation by hearing devices. Even cochlear implantation and bone-anchored hearing aids could radically improve the quality of life of patients with SNHL, but technological advances are needed to restore hearing by participation in regeneration of neural and cochlear tissue [44].

The discoveries made over the past decade have allowed the development of cell therapies to protect, restore, and regenerate the hearing system and treatment of hearing loss and deafness [45]. In regenerative therapies, cells such as neurons, HCs, and spiral ligaments (SLs) are promising targets in the inner ear treated by transplantation of various stem cell types, such as ESCs, iPSCs, and MSCs. There is a high risk of rejection and tumor formation in iPSCs and ESCs transplantation; MSCs lack these drawbacks and were exhibited to be beneficial in treatment of inner ear inflammatory damages, based on their low immunogenicity, multidirectional differentiation potential, and immunosuppressive function [46].

Current findings on MSCs treatment potential in otorhinolaryngology are mostly based on animal models. Since cells with stem/progenitor properties appear to be no longer present in the mammalian cochlea three weeks after birth, application of exogenous MSCs and their differentiation into the missing auditory cells has opened new opportunities in sensorineural diseases and inner-ear cochlear dysfunction. MSCs differentiated to neurosensory progenitors were shown to express the markers of early otic development including nestin, orthodenticle homeobox 2 (Otx2), Sox2, Brn3c, GATA binding protein 3 (GATA3), Musashi, the early HC genes Math1, and the sensory neuronal markers such as tropomyosin receptor kinase B (TrkB) and TrkC [47,48].

It should be noted that HCs are not easily produced by stem cells, and the insertion of the implanted cells into the organ of corti can be blocked due to the high potassium level of the endolymph, the hostile environment of the cochlea itself, and the presence of tight junctions, so the survival of the transplanted cells is not guaranteed [49]. In this relation, administration of exogenous stem cells into the inner ear was demonstrated to replace injured hair cells and/or neurons [48,50]. Two approaches for the delivery of exogenous stem cells have been reported, including into the scala tympani through the round window, or cochleostomy, and inducing them to migrate into the organ of Corti; direct injection into the scala media and optimizing the survival of the transplanted cells [51]. The differentiated cells could interact with hair cells and auditory neurons of cochlear explants via formation of new synapses [52]. Current challenges in stem cell transplantation are cell survival and differentiation after transplantation, especially in the scala media of the cochlea, which contains high potassium endolymph. Proper arrangement of sensory cells is vital because engraftment of supernumerary hair cells in the cochlea does not restore function [14].

Various animal models, such as zebrafish, frog, birds, and mammals, have been used to investigate the human inner ear function, diseases, and treatment measures because access to human inner ear tissues is strictly limited, and tissue sampling is technically challenging and with irreparable injuries [53]. It is necessary to mention that both mammals and humans do not possess the innate ability to regenerate the lost sensory cells in the cochlea when development is completed [54]. There are limited numbers of stem cells in the cochlea possessing limited proliferation potential to express the markers of adult stem cells too [55]. Non-invasive procedures, such as computed tomography (CT) scan or magnetic resonance imaging (MRI), cannot provide enough resolution to determine most pathologies of the inner ear [53]. Therefore, transplantation of exogenous MSCs can take advantage of an array of stem cells from various tissue sources [4].

The first evidence of mammalian inner ear stem cells (IESCs) dates from 2003 and were obtained, specifically, from the utricular macula, so they could be differentiated into ciliated cells in vitro and in vivo [56]. Differentiation of MSCs to hair cell progenitors was also first reported in vitro by Jeon et al. in 2007 [57]. There are still challenges in cell transplantation therapies, including their differentiation to sensory cells without contamination with other cell types, that may interfere with cell transplantation and organ function, their survival and integration into the cochlear sensory epithelium and finally, the technical challenges of cell delivery to the damaged tissue, although a surgical approach may be applied to expose the cochlea for implantation or injection of therapeutic cells into the tissue [58].

Duran Alonso et al. were the first who differentiated human bone marrow-derived stem cells (BMSCs) into neural progenitors and then, induced sensory neuron phenotype via supplementation of the medium culture with SHH, retinoic acid, brain-derived neurotrophic factor (BDNF), neurotrophin-3 (NT-3), and bFGF [59]. Jakob et al. successfully identified MSCs from the nasal mucosa with similar characteristics to classical BMSCs, ability to divide rapidly to differentiate into adipocytes, osteocytes, and chondrocytes and to be plastic adherent [60]. BMSCs were demonstrated to possess the capacity to differentiate into auditory neuron-like cells in vitro in the presence of BMP4 by up-regulating the inner ear specific genes, such as neurofilament medium polypeptide (NF-M), neurog1, gluR4, neuroD, calretinin, neuN, tau, and GATA3 [61]. Human BMSCs differentiation into an intermediate neural progenitor stage to obtain inner ear sensory lineages, hair cell-like cells and auditory cells in serum-free medium containing epidermal growth factor (EGF) and retinoic acid was previously shown [59]. BMSCs could differentiate into neuron-positive and hair cell-positive cells that could have a promising effect in the SNHL of rats [62]. Buddy et al. have successfully forced human BMSCs in vitro to express essential genes in the otic lineages [63]. When BMSCs, in combination with growth factors that were positive for expression of the transcription factor Atoh-1 were utilized, differentiation into hair cells was shown [57]. The safety of an intravenous injection and transplantation of autologous BMSCs has been confirmed in two patients with SNHL [64]. Lin et al. could reprogram murine AdSCs into hair cell progenitors using a combination of protein transfection, adenovirus, and co-culture with neurons [65].

## 8. In Vivo Studies of Inner Ear Hearing Loss

Table 1 presents in vivo studies of inner ear hearing loss based on stem cell source and year of study. Several types of stem cells, including BMSCs, AdSCs, umbilical cord stem cells (UCSCs), tongue-derived stem cells (TSCs), olfactory epithelium neural stem cells (oeMSCs), nasal tissue-derived stem cells (NSCs), and hematopoietic stem cells (HSCs) have been utilized in the treatment of inner ear hearing loss in animal models of mouse, rat, gerbil, and Guinea pig. There are many studies regarding the use of BMSCs in treatment of inner ear hearing loss. Li et al. used BMSCs in rats and observed cell transformation into neuron-like cells and positive expression of neurofilament (NF-200), microassociate protein-2 (MAP-2), neuron-specific nuclear protein (NeuN), nestin, gial fibrillary acidic protein (GFAP), GAD, and ChAT by immunohisitochemistry [66]. Intravenous transplantation of BMSCs and HSCs in mice resulted in integration of cells in the cochlea, and their differentiation, to inner ear fibrocyte-like cells without any adverse effects on auditory function [67]. Perilymphatic transplantation of green fluorescent protein (GFP) transgenic mouse BMSCs in the gerbil model of auditory neuropathy (deafened with ouabain) via scala tympani or modiolar injection demonstrated survival of MSCs within the modiolus that participated in the regeneration of damaged SGNs without any evidence of hyperacute rejection [68]. Transplantation of BMSCs into lateral semicircular canals in the rat model of acute SNHL, secondary to fibrocyte dysfunction (mitochondrial toxin), resulted in detection of MSCs in injured lateral cochlear wall, expression of connexin 26 and connexin 30, reactivation in gap junction between neighboring cells, an increase in cell survival, and acceleration of hearing recovery through the repair of injured cochlear fibrocytes [69]. Ratajczac et al. utilized BMSCs in mice and noted very small, embryonic-like cells with the potential to develop into neural and other cells for tissue repair [70]. GFP transgenic mouse BMSCs transplantation into the perilymphatic space of normal cochleae in mice displayed that transplanted cells could settle within the cochlear tissues, especially in the SLs and the spiral limbus, although most transplants were located in the perilymphatic space. Some of the transplanted cells expressed the cochlear gap-junction protein connexin 26, indicating their potential for restoration of cochlear cells [71].

Tan et al. in autoimmune deafened adult Guinea pig by use of IL-4-expressing BMSCs into scala tympani, via lateral wall, reported homing and survival capability of cells to the deafened cochlea, and transdifferentiation of them to any cochlea cell types [72]. Cho et al. utilized human BMSCs in vitro neural differentiated cells into the scala tympani to treat a Guinea pig animal model with ouabain-induced auditory neuropathy, leading to an increase in SGNs number and improvement of hearing function [73]. Intravenous injection of human BMSCs in a rat model of noise-induced or ototoxic SNHL resulted in survival of a small number of MSCs within the spiral ganglion area while most MSCs were trapped in the lungs [74]. In the Guinea pig model, the potential of AdSCs and BMSCs differentiation into neuron-like cells was demonstrated [75]. In mice, hearing recovery, after transplantation of BMSCs into the inner ear, was shown to happen, due to transdifferentiation of BMSCs into sensory fibrocyte-like (SFL) cells and stimulation of host SFLs regeneration [76].

Mouse MSCs transplanted into ampulla of superior semicircular canal in young and old healthy mice could show migration of BMSCs to cochlea, and their differentiation into SFL cells, in young mice without any adverse effects on auditory brainstem response (ABR) [77]. Human BMSCs transplanted into neomycin-deafened Guinea pig cochlea led to an increase in number of SGNs in organ of Corti and spiral ganglion, and their differentiation into neuronal progenitor cells and neuronal cells, to be used in treatment of SNHL, based on the induction of stem cell homing factors in the host cochlear tissue [78]. Magnetic labeled MSCs have been investigated in otorhinolaryngology too. To track the condition of cells transplanted in the inner ear, superparamagnetic iron oxide nanoparticles (SPIONs) have been incorporated into BMSCs, and the cells were injected to the inner ear of Guinea pig and monitored by a 1.5 Tesla MRI (Siemens, Munich, Germany) to confirm that the stem cells were successfully engrafted in the inner ear [79]. Mahmoudian-Sani et al. suggested that BMSCs, in comparison to AdSCs and UCSCs, had more efficacy to migrate and survive in the cochlear tissues, regenerating inner ear, and treating SNHL [80].

In mice, BMSCs were shown to enhance the regeneration and maintenance of fibrocytes in damaged SLs, leading to partial restoration of cochlear function [81]. Transtympanic transplantation of rodent BMSCs in a non-immunocompromised rat model to assess cochlear function by ABR, distortion product otoacoustic emissions (DPOAE), and histopathology did not reveal the recruitment of inflammatory leukocytes and edema in the cochlea of MSCs administered rats [82]. Fetoni et al., in a noise deafened adult Guinea pig, found that transplanted AdSCs into scala tympani, via round window, were able to survive and migrate, at the site of tissue damage, and express trophic factors to pave the way for further treatment of deafness [83]. Intraperitoneal injection of human AdSCs, two weeks after the onset of hearing loss, in mouse model of experimental autoimmune hearing loss (EAHL) was treated with β-tubulin, demonstrated immunomodulatory properties, absence of atrophy in stria vascularis or organ of Corti, and improvement in hearing function, presented as decreased thresholds of ABR, and protected HCs in established EAHL [84].

Injection of magnetically labeled AdSCs into the cochlea of kanamycin deafened rats revealed elevations of BDNF, the presence of significant number of MSCs in the cochlea, improved survival of SGNs, and improved hearing threshold levels denoting to their protective effects against loss of auditory function [85]. Intravenous transplantation of intact human UCSCs in a deaf Guinea pig model revealed an improvement in hearing thresholds via relocation and a rise in the number of SGNs [86]. Sullivan et al., in an adult mouse deafened by noise, demonstrated that administration of mouse TSCs into scala tympani/scala vestibulivia lateral wall led to an increase in cell survival and could attenuate the ototoxic effects of noise trauma [87]. The administration of oeMSCs into the mice cochleae, with lateral wall cochleostomy hearing loss, exhibited survival of implanted stem cells within the perilymphatic spaces of the scala tympani and conservation of hearing with otoprotective activity of oeMSCs, via stimulation by native spiral ligament fibrocytes and transdifferentiation into SFLs and stimulation of regeneration in situ of host spiral ligament fibrocytes, from a resident stem cell population by paracrine nature of MSCs [88].

Direct injection of rat oeMSCs, into the cochlear of noise-induced hearing loss model of rats, resulted in migration of stem cells around the spiral ganglion neurons and restoration of hearing loss after cell implantation, as assessed by ABR [89]. Injection of human olfactory stem cells (OSCs) into cochleae of mice, via lateral wall cochleostomy, with early onset progressive SNHL resulted in a reduction in inflammation, oxidative stress, cell apoptosis, a significant lower hearing threshold level, and amelioration of hearing loss [90]. The human MSCs, derived from nasal tissue, were evidenced to repair spiral ganglion loss in experimentally injured cochlear of neonatal rats via direct neuronal differentiation and secondary effects on endogenous cells [91] that can be tracked and verified, in a non-invasive manner, after cell transplantation by using MRI contrast agents [79,85,92].

## 9. Tympanic Membrane Hearing Loss

Chronic otitis media is the primary cause of conductive hearing loss that involves perforation of the tympanic membrane (TM) and erosion of the ossicles. The TM, or eardrum, is a thin, protective layer of middle-ear tissue that forms a boundary between the external and middle ear. It is consisted of three main parts of larger pars tensa, the smaller pars flaccida, and umbo that are also other components of the hearing process in the auditory system. The anatomical structure of the TM has three layers of ectoderm, mesoderm, and endoderm [46].

TM is responsible for amplifying and transmission of sound vibrations through a chain of mobile ossicles and its perforations. External sound pressure, middle ear infection, severe trauma, and insertion of sharp objects into the ear can lead to deficient hearing function. Three primary issues in the repair of TM perforation have been reported including absence of structural assistance, absence of extracellular matrix, leading to weak neomembrane adhesion of cells, and limited angiogenesis and growth factors [93]. It is necessary to mention that wound healing in TM is slightly different from wound healing in other cutaneous tissues [94], so an exudate is released around the edges of the perforated TM, after injury, which can protect the tissue from dehydration and facilitate cell migration and proliferation of stratified squamous epithelial layer to the perforation center [95].

To close the perforated eardrum, surgical procedures (myringoplasty or tympanoplasty) are undertaken by otorhinolaryngologists, but limitations, such as discomfort, side effects, and high cost of surgical treatment have necessitated the use of better alternatives, such as tissue engineering and MSCs transplantation, as a promising tool to overcome the limitations, the operational risks and to restore, to maintain, and to improve the TM function [46]. In an injured eardrum, cell-based therapies have opened a way to solve these limitations, because MSCs can migrate towards the site of injury and participate in cell survival, cell proliferation, and tissue angiogenesis by secretion of trophic factors such as vascular endothelial growth factor (VEGF), EGF, IGF, hepatocyte growth factor (HGF), nerve growth factor (NGF), TGF-α, and stromal derived factor-1 (SDF-1), along with chemoattractant gradients in the stromal extracellular matrix and peripheral blood [96], where local factors such as hypoxia, toll-like receptor ligands, and the cytokines activate the MSCs to foster the entry of more growth factors to boost tissue regeneration [97].

In this relation, MSCs in the TM must have an appropriate microenvironment to facilitate cell survival and proliferation. If, during introduction of MSCs in the perforated TM, the cells are dropped into the middle ear cavity, the cells would be easily susceptible to air-drying through external auditory meatus [98]. Another important point in cell transplantation is the cell delivery that uses of scaffolds as an increasingly popular technique that can provide protection and controlled spatial cues for seeded stem cells. The delivery of MSCs at the ruptured TM sites was shown to enhance the activation of epithelial stem cells for faster closure of TM perforation. So, the fibroblasts and collagen in the middle connective tissue layer produce a neomembrane framework to close the perforation [99]. Danti et al. have fabricated colonization of human MSCs on scaffolds to allow an osteoblastic maturation in vitro [100].

## 10. In Vivo Studies of Tympanic Membrane Related Hearing Loss

Rahman et al. used drops of gelatin, containing human BMSCs, on the perforated TM of rats and assessed the thickness of pars tensa region and lamina propria under otomicroscopy, mechanical stiffness of the healed TM tissue by Moiré interferometry and lamina propria, middle ear cavity, and external ear canal wall tissue via microscopy and illustrated a decrease in the stiffness of the healed tympanic membrane and a healing process with an enhanced restoration [101]. When GFP expressing BMSCs were embedded in porcine-derived (Gelita-Spon GS), hyaluronate-derived (EpiDisc ED), and polyvinyl alcohol (PVA) scaffolds and injected in an injured TM of a mouse model, the transplanted cells were deposited in the injured tissue and differentiated into epithelial-like cells and formed a thicker neotympanum that can be a promising alternative to tympanoplasty [102].

The first animal model trial of concurrent use of MSCs and a three-dimensional (3D) bioprinted scaffold (polycaprolactone/collagen/alginate) was carried out in closing of subacute TM perforations in rats undergoing otoendoscopy for acoustic measurements as per ABR thresholds. The findings denoted to the recovery of the hearing capacity at all frequencies, along with regeneration of the thick neodrum assessed by optical coherence tomography (OCT). Goncalves et al., by use of BMSCs seeded on hyaluronic acid (HA) scaffold in mice bilateral large tympanic perforations, demonstrated the repair in TM and restoration of the trilaminar structure in TM. Assessment by histology indicated formation of an intact neotympanum in the perforated areas. Neo-tympanal integrity and transparency were confirmed by otoscopy too [103]. The mechanical properties of the regenerated TM, such as membrane stiffness, membrane stability, and efficient nanovibration were evaluated by a laser Doppler vibrometer (LDV), revealing an acceleration in the healing process in the TM perforations and formation of a thickened prominent fibrous layer. The acoustic mechanical properties were recovered in the healed TM [104]. Ong et al. found that human AdSCs in mice with sub-total pars tensa perforations could lead to paracrine function, secretion of growth factors, a promoted significant keratinocyte proliferation and migration and TM wound healing [105]. Table 2 presents in vivo studies oftympanic membrane related hearing loss based on stem cell source and year of study.

## 11. Larynx and Vocal Cord: Larynx

The larynx is a dynamic organ with considerable complexity that should be considered when laryngeal reconstruction is targeted, as a neo-larynx needs functional muscle tissue with appropriate re-innervation. Therefore, decellularized skeletal muscle matrices are utilized as a potential scaffold for production of the muscular activity required for an engineered larynx [106]. Impairment in laryngeal function related to vocalization, swallowing, and respiration can be life challenging and devastating. Problems with swallowing, taste, speech, smelling, breathing, lifting, and aesthetic appearance can lead to a substantial impairment of quality of life, and can affect social functioning and the ability to work. To treat stenotic airway, especially in the subglottic area of laryngotracheal defected patients, laryngotracheoplasty is undertaken, which involves the use of cartilage interpositional grafting. Although a total laryngeal transplantation and replacement would significantly improve the quality of life for these patients, but problems associated with this procedure requiring life-long immunosuppression represent a major ethical question and limitation for the procedure [107].

Among laryngotracheal defects, laryngotracheal stenosis is the most often encountered case with considerable morbidity and mortality that happens congenitally or acquired after prolonged intubation and hypertrophic scarring, and is associated with narrowing of the airway at larynx, subglottis, or trachea [108]. Current choices in treatment of the stenosis are laser surgery, endoscopic dilation, laryngotracheal reconstruction, or life-long tracheostomy, but they can result in formation of new scar tissues and a further restenosis [108]. The regenerative medicine approach, by using MSCs and scaffolds, can represent a significant advantage over these limitations in otorhinolaryngology clinical practices [107].

Transplantation of MSCs was shown to be effective in regeneration of a functional laryngeal tissue and help restoration to a normal anatomy, especially when bioengineering is added to cell therapy that can provide a larger surface area to promote tissue regeneration and increase tissue function [109]. Significant advances have been observed in the generation of stem cell derived airway grafts, construction of a tissue-engineered larynx, and in laryngotracheal stenosis [110] because cell transplantation has anti-inflammatory and immunosuppressive properties, possesses the ability for cell migration to the exact area of injury, and has the potential to secrete soluble factors that are vital for cell survival and proliferation. Cell-based therapies were shown to have minimal side effects and are easily accessible for isolation too [111]. A seeding density exceeding 1 × 10^6^/cm^2^ was illustrated to be an appropriate number of transplanted cells to accelerate the tissue integration process and activate local progenitor cells [112].

## 12. In Vivo Studies of Larynx

Jotz et al. compared laryngeal defect closure in a porcine model using a naïve nanofiber scaffold seeded with dental pulp stem cells (DPSCs) displaying a significant advantage with formation of neocartilage tissue [113]. Ansari et al. indicated that implantation of a de-cellularized hemi-larynx seeded with human BMSCs in pig models could allow for vascularization and further orthotopic implantation without any adverse effect on respiratory function, swallowing, or vocalization. Rudimentary vocal folds, covered by contiguous epithelium, were also identified [114]. Iravani et al. used BMSCs in laryngotracheal stenosis in a dog model and found a complete epithelialization with minimal chronic inflammatory cell infiltration in the submucosa of vocal folds [115]. Herrmann et al., in implantation of a tissue engineered BMSC in a pig model, found an appropriate mucosal coverage and rudimentary vocal fold development, without any adverse effect on respiratory function, swallowing, or vocalization [107]. Table 3 presents in vivo studies of laryngeal defects and disorders based on stem cell source and year of study.

## 13. Larynx and Vocal Fold: Vocal Fold

Based on Hirano’s body-cover theory, the vocal folds are consisted of a superior layer (“cover”) including epithelium, basal membrane, and the superior part of the lamina propria and an inferior layer (“body”) with deep lamina propria and thyroarytenoid muscle being, separated by an intermediate layer of lamina propria. This architecture causes these two functional units to vibrate independently and is found in the mid-part of the vocal folds; the anterior and posterior areas, which are the site of maculae flavae, illustrate a different architecture, which functions as a buffer [116]. After laryngeal microsurgery, vocal fold microstructure and scarring can happen, due to partial disappearance of the lamina propria, with the superficial and/or intermediate layer changed by fibrous tissue, inhibiting mechanical uncoupling of the epithelium and muscle and thereby inducing vibration disorder and disabling dysphonia [117].

Treatment choices, presently, are few, and mostly without efficacy for vibration, posing just an effect on volume to decrease glottal closure defect. So, in the current state of the literature, cell transplantation has been introduced in vocal fold scarring [118]. Chen and Thibeault, in an in vitro study, co-cultured healthy and scarred vocal fold fibroblasts, with BMSCs added to HA hydrogel, and reported an inhibition of fibroblast proliferation without any effect on morphology or viability [119]. Hiwatashi et al., in an in vitro study, investigated the effect of TGF-1 expression by co-culturing normal vocal fold fibroblasts with AdSCs or BMSCs in presence or absence of TGF-1 and found that MSCs could regulate extracellular matrix composition by a decrease in type I and III collagens, inhibited TGF-1 expression, and differentiation toward myofibroblasts, by a decrease in smooth muscle alpha-actin (α-SMA) levels [120]. Kumai et al., in two in vitro studies using ferret AdSCs, showed that when fibroblasts, co-cultured with AdSCs, proliferated less and expressed less α-SMA, less collagen, and more HA and HGF [121,122].

## 14. Clinical Trials of Vocal Fold Scarring

Karolinska University Hospital, in phase I clinical trial in an open labeled single-group of sixteen 18 years and older patients with severe hoarseness and vocal fold scarring evaluated the injection of autologous BMSCs with a hyaluronan gel. The safety, efficacy, healing process including inflammation, polyp/granuloma formation, and vascularization were assessed. Functional measures, including high-speed imaging, acoustic voice analysis, and phonation pressure measurement were evaluated. The outcome was improved healing of scarred vocal fold, one year postoperatively [123]. Assistance Publique Hopitaux De Marseille in an open labeled single-group clinical trial enrolling eight 18 years and older patients with vocal fold scarring and dysphonia injected autologous AdSCs and reported the feasibility, safety, and efficacy of the procedure and functional measures of voice handicap index evaluated by laryngostroboscopy [124]. Lo Cicero et al. confirmed use of AdSCs in patients who had undergone vocal fold lipoinjection with laryngeal hemiplegia or defects and demonstrated the therapeutic efficacy of this clinical approach and restoration of glottic competence [125]. Table 4 represents clinical trials in treatment of vocal fold scarring using MSCs based on stem cell source and year of study.

## 15. In Vivo Studies of Vocal Fold Injuries

Kanemaru et al. utilized BMSCs with atelocollagen in injured vocal fold of dogs and exhibited that 3D incubated BMSCs were beneficial in regeneration of the injured vocal fold [126]. BMSC-based therapy in GFP transgenic mice with resected vocal folds could improve the quality of the healing process in vocal fold injuries [127]. Hertegard et al. in scarred rabbit vocal folds after injection of human BMSCs indicated to improved viscoelastic parameters and less signs of scarring expressed as collagen content [128]. Johnson et al., in scarred vocal fold lamina propria of a rat model, transplanted BMSCs with decellularized scaffolds, in combination with growth factors, found the most favorable outcomes in ECM production, graft survival, myofibroblast differentiation, hyaluronan metabolism, production of TGF-β1in, absence of any cytotoxicity, and preservation of local cell proliferation [129]. Svensson et al., in scarred vocal folds of rabbits, injected BMSCs and noted an improvement in healing of the vocal fold injury with reduced lamina propria thickness and collagen type I content and restoration of viscoelastic function [130].

In rabbits with transplanted BMSCs into the vocal fold scar, MSCs could enhance the functional healing of the vocal fold with decreased lamina propria thickness and restored viscoelastic shear properties [131]. Ohno et al. served BMSCs and atelocollagen in vocal fold scarring of a dog animal model and displayed an increased HA distribution and a decreased dense collagen deposition in the lamina propria that could lead to a better mucosal vibration [132]. Kim et al. xenografted mouse BMSCs in a rabbit vocal fold injury and demonstrated cell survival in the injured xenogeneic vocal folds after cell transplantation with favorable and enhanced wound healing in vocal folds [133]. Hiwatashi et al., in a rat model of vocal fold scar regeneration, used GFP-labeled AdSCS and BMSCs with HA and showed the regenerative effects of AdSCs and BMSCs transplantation in vocal fold scars. The regenerative effects of AdSC and BMSC transplantation were found to be identical. bFGF2, HGF, and Has3 were upregulated in both cell transplantation groups. AdSCs seemed to upregulate HGF more than did BMSCs [134].

Choi et al. injected BMSCs with porcine gel in scarred vocal folds of a rabbit model and suggested the complex as a plausible biomaterial for prolonged survival of BMSCs in vocal folds to promote scarless vocal fold healing [135]. Bartlett et al., for restoring voice in rabbits with vocal fold scarring, used BMSCs and verified early resolution of viscoelasticity, a decrease in inflammation and facilitation of tissue repair [136]. Lerner and colleagues injected BMSCs intravenously after recurrent laryngeal nerve (RLN) injury in the rat and observed complete recovery of vocal fold mobility and functional recovery [137]. Lee et al., in a dog animal model, used AdSCs together with atelocollagen into injured vocal fold and illustrated multipotential ability of AdSCs in regeneration of injured tissue and their preventive activity in vocal fold scarring and atrophy [138]. Kwon and Lee demonstrated that transplantation of AdSCs, with HA and HGF, into injured vocal fold of rabbits could prevent vocal fold atrophy after laryngeal surgery [139]. Nishio et al. investigated the efficacy and safety of AdSCS in a pig unilateral vocal fold paralysis model and showed a noticeable hypertrophy in thyroarytenoid muscle fiber in the injection site with improvement in unilateral vocal fold paralysis [140].

Xu et al., in a rabbit vocal fold wound model, administered AdSCs together with collagen or HA and found their facilitatory role in vocal fold regeneration, regulating the generation and orderly distribution of extracellular matrix (ECM) [141]. Hong et al. injected human AdSCs in injured vocal folds of a rabbit model and observed a decreas in collagen content in the treated folds, fewer signs of scarring, an increase in viability of stem cells in vocal folds, and an improvement in wound healing [142]. Liang et al. denoted vocal fold regeneration when AdSCs were used in a rabbit acute vocal fold injury model [143]. Kim et al., in vocal fold wound of rabbit model, showed that injection of AdSCs seeded on alginate-HA hydrogel resulted in prolongation of the retention time of stem cells in the vocal folds and a promotion in wound healing [144]. Hu et al., in a dog model of acute vocal fold wound, transplanted AdSCs and showed the ability of cells to secrete ECM, particularly elastin, which may be beneficial for vocal fold vibration recovery components and also an improvement in vocal fold wound healing [145]. Shiba et al., in rabbits, evaluated the impact of AdSCs and fibrin hydrogel on phonatory function and wound healing of vocal folds and demonstrated minor evidence of scar formation and immune reaction after transplantation. Vibration was preserved and a complete reconstruction in vocal fold cover layer was noted [146].

Angelou et al. used autologous AdSCs in chronic vocal fold scar in a rabbit model and showed enhanced healing in vocal folds and a reduction in scar tissue [147]. De Bonnecaze et al. administered AdSCs in severe vocal disturbance of an acute vocal fold scar in rabbits and realized an improved vocal fold healing [148]. Valerie et al. revealed that use of autologous AdSCs, in a chronic vocal fold scar in a rabbit model, could enhance the healing of the vocal fold injury and the reduction in the scar tissue [147]. Halum et al. used rat muscle stem cells in vocal fold paralysis and demonstrated myoblast survival with attenuation of muscle atrophy after cell transplantation [149]. Halum et al., in another study, transplanted muscle stem cells in rat vocal fold paralysis and reported enhancement of MSC survival and promotion of neural regeneration [150]. Halum and colleagues in rats underwent RLN transection injury, injected muscle stem cells and demonstrated an enhanced reinnervation state [151]. Peng et al. in dog model of vocal fold injury found that laryngeal stem cells (LSCs) differentiated into myofibroblasts and fibroblasts and could regulate extracellular matrix, inhibit the rapidly decrease in elastic fiber and HA, decrease the microenvironment inflammatory reaction, block collagen and the fibronectin rapid increase, and prevent the formation of vocal fold scar [152]. Table 5 presents in vivo studies of vocal fold injuries based on stem cell source and year of study.

## 16. Nose and Paranasal Sinuses

The nose and paranasal sinuses in adults cover an approximate surface of 100–200 cm^2^ and are lined with pseudostratified columnar ciliated epithelium that has important physiological functions such as inspired air conditioning and filtration, while about 10 to 20,000 L of air move daily through the nasal cavities to the lungs. Nose serves as an end organ for the sense of smell and plays an important role as a physical and immunological barrier for interaction between the host tissue and foreign invaders (allergens, bacteria, and viruses) [153]. In the healthy nose, more than 90% of small particles in the inhaled air are trapped on the nasal mucosa surface and are transported to the pharynx, where they are either swallowed or coughed up by the mucociliary apparatus [154]. The nasal septal mucosa is rich in chondrogenic cells and the olfactory epithelium has mesenchymal properties [155]. The mucosal lining of the nasal cavity is covered with goblet cells, in the epithelium, and submucosa seromucous glands that can produce 100–200 mL of mucus over 24 h in a resting rate [156]. The olfactory system, as an extracranial part in the nasal mucosa, retains regions of olfactory epithelium with endogenous population of stem cells that allows harvesting without any damage to the donor [157].

In the nasal skeleton, cartilage tissue is avascular, developed from cranial neural crest cells of the mesectoderm, and its main function is to shape the nose. It can be easily provided by septoplasty for cartilage tissue engineering purposes and has the potential for production of fibrocartilaginous tissue to satisfy the needs for structural support and graft durability for repair and replacement of the damaged cartilage, which is the most useful building block for rhinoplasty and in severely deformed noses [36]. Based on interior structure and specific shape of the human nose, the aerodynamics of airflow alters significantly from a laminar flow at the vestibule to a turbulent flow, anterior to the head of the inferior turbinate facilitating mucosal contact for humidification, heating/cooling, and filtration of inspired air [158]. A previous experience benefited from auricular concha cartilage or rib cartilage for rhinoplasty and deformed noses; as costal cartilage has the warping problem, and auricular cartilage is not a proper choice for the struts of axial nasal structures; they cannot satisfy the requirements for graft materials [159]. So current reconstructive and augmentative rhinoplasty surgeries using autologous tissue grafts to repair nasal trauma or attain an aesthetic shape are associated with donor site trauma and morbidity [160].

Synthetic materials have been used for repairing nasal trauma, but they can yield an unnatural appearance and are prone to infection or dislocation. Chondrocytes provided from fully mature nasal septum cartilage are the other alternative to reconstitute the nasal alar lobule and to repair articular cartilage defects with regenerative properties that has warranted their translation into clinical scenarios [160]. As chondrocytes have limited proliferation potential and tendency to dedifferentiate, addition of MSCs for cartilage tissue engineering in regenerative medicine has become one of the most intriguing candidates without undesirable features in rhinoplasty and rhinosinusitis [160].

Rhinosinusitis is a common nasal disease that affects approximately 5–15% of the general population and the quality of life of the patients [161]. Treatments such as antibiotics, steroid, nasal douche, nasal sprays, and endoscopic sinus surgery are usually administered to patients with rhinosinusitis to reduce inflammation, eliminate infection, and to revert the diseased mucosa to normal function, but the respiratory epithelium does not undergo restoration and amelioration. Therefore, in rhinosinusitis, regenerative medicine by employing a multidisciplinary approach of achieves tissue repair using stem cells, scaffolds, and bioactive molecules [162].

## 17. Clinical Trials of Diseases in Sinuses

Shayesteh et al. in a clinical trial used BMSCs in combination with biphasic hydroxyl apatite/ β-tricalcium phosphate (HA/TCP) for sinus elevation and reported cell transplantation as a viable therapeutic alternative for implant placement [163]. Rickert et al. in a randomized controlled trial in 12 consecutive patients with atrophic maxilla and bilateral sinus floor augmentation used BMSCs, seeded on bovine bone mineral (BioOss), and demonstrated formation of sufficient volume of new bone as an alternative to using autografts to enable the reliable placement of implants within a time frame [164]. Gonshor et al. assessed bone formation in a 40-year-old female patient with maxillary injury following sinus-augmentation procedures using either an allograft cellular bone matrix, containing native BMSCs and osteoprogenitors, or conventional allograft, and revealed a high percentage of vital bone content, after a relatively short healing phase, a rapid initiation of implant placement or restoration when cell transplantation was conducted [165]. Wildburger et al. in seven patients with bilateral highly atrophic posterior maxilla showed that transplanted BMSCs or pure bovine bone material did not have any significant difference in new bone formation [166]. Kaigler et al. in a phase I/II randomized, controlled clinical trial assessed the reconstruction of bone deficiencies of the maxillary sinus by implanting autologous BMSCs enriched with CD90+ stem cells and CD14+ monocytes and found the therapy to be safe for maxillary sinus floor reconstruction and to accelerate and enhance the tissue engineered bone quality [167]. Pasquali et al. in a randomized controlled trial in eight consecutive patients undergoing sinus floor lift procedures with Bio-Oss alone or combination of BMSCs and Bio-Oss showed an increase in bone formation in sinus lift procedures when BMSCs were utilized [168]. Table 6 presents clinical trials in treatment of sinuses diseases based on stem cell source and year of study.

## 18. In Vivo Studies in Rhinology

Sun et al., in a mouse model of ovalbumin-induced allergic inflammation in upper and lower airways, assessed the impact of systemic administration of human iPSC-MSCs and BMSCs and demonstrated a decrease in inflammatory cell infiltration, a decrease in serum levels of Th2 immunoglobulins and cytokines in bronchoalveolar and/or nasal lavage fluids and their protection from allergy-specific pathological changes [169]. Kwon et al., in a rat model of olfactory nerve degeneration, reported use of BMSCs to accelerate regeneration of olfactory mucosa [170]. BMSCs have been successfully used with chondrocytes and cellulose and alginate for chondrocyte proliferation and cartilage formation in mice [171]. The coculture of human nasal septal chondrocytes and BMSCs, and their transplantation implanted in the immunodeficient athymic nude mice model, resulted in synergistic cartilage matrix production, termination of tissue calcification, and generation of a stable implantable 3D engineered cartilage graft [172]. Kim et al., when investigating the effect of intravenous injection of AdSC in unilateral transection of the olfactory nerve and degeneration of olfactory epithelium, demonstrated restoration of the thickness and cellular composition of epithelium, differentiation into olfactory receptor neurons and endothelial cells, and a promoted regeneration in olfactory epithelium [173].

Cho et al. found that AdSCs could migrate to the nasal mucosa in an allergic rhinitis mouse model and could inhibit eosinophilic inflammation partly via shifting to a Th1 from a Th2 immune response to allergens [174]. Kavuzlu et al. implanted AdSCs onto the nasal mucosa in the nasal injury rabbit model and reported abundance, and density, of the ciliated nasal epithelial cells after transplantation and enhancing of the tissue healing [175]. In a model of allergic rhinitis in mice, induced by ovalbumin intraperitoneal injection and the nasal stimulation induction method, GFP-labeled human UCSCs when injected intraperitoneally or via tail vein could reach the nasal cavity, inhibit the expression of the cytokines IL-10 and INF-γ, and prevent allergic responses [176]. De Corgnol et al. used olfactory ensheathing cells, in rats with vagus nerve section, and reported the reinnervation in the vocal folds [177]. Transplantation of tonsil-derived stem cells in mice allergic rhinitis model showed significantly decreased allergic symptoms, a reduced infiltration of eosinophils and neutrophils in the nasal mucosa and a significantly declined IL-4 mRNA expression that can demonstrate the immunomodulatory effect of tonsil-derived stem cells via inhibition of T cell activation, mitogen-activated protein (MAP) kinase, p65, and NFAT1 [178]. The intravenous injection of tonsil-derived stem cells, in a model of allergic rhinitis in mice, induced by ovalbumin, showed a significantly reduced allergic symptoms, a decrease in eosinophil infiltration, a decline in total serum and immunoglobulin E (IgE), a reduction in the nasal and systemic Th2 cytokine profile, and finally, an inhibition in allergic inflammation [179]. Table 7 demonstrates in vivo studies in rhinology based on stem cell source and year of study.

## 19. Head and Neck Surgeries

Craniofacial bones are derived from the cranial neural crest and are flat and develop mainly through intramembranous and endochondral ossification. They have little marrow and are sheathed by periosteum and dura [180]. Defects of the soft and hard tissues in the maxillofacial region can severely impair oral functions and cosmetic appearance, thereby leading to many dilemmas in craniofacial surgery [181]. Craniofacial surgery, since its inauguration, has been the culmination of collaborative efforts to solve complex congenital, oncological, dysplastic, severe infection, mental health, and traumatic cranial bone defects. One of the major concerns in maxillofacial surgery is finding a procedure to improve regeneration of large craniofacial bone defects, while the need for bone repair due to aging of the population has increased too [182]. There are many drawbacks in craniofacial surgery, such as the morbidity of the donor site and the limited number of transplanted tissues that can be considered as limitations in clinical practice [183].

Nowadays, regenerative medicine, by utilization of various stem cells including ESCs, iPSCs, and MSCs, has opened a new window in treatment of craniofacial defects and diseases based on anti-inflammatory, immunomodulatory, and antiapoptotic properties of MSCs. Their osteogenic potential, and lack tumorogenic activities, being seeded easily onto scaffolds have also resulted in successful use of cell therapy and tissue engineering for reconstruction of craniofacial defects [184], even the influence of the niche environment and ECM on stem cell fate, behavior, and ability to undergo differentiation towards a specific lineage, such as fat, bone, and cartilage and vascularization in craniofacial defects is of great importance [185]. The behaviors in MSCs to undergo migration, proliferation, differentiation, and angiogenesis are mediated by various cytokines. When MSCs are derived from young donors, they have a higher expression of osteogenic markers, such as osteopontin, osteocalcin, and BMP-2, and a higher content of mineral calcium deposits that can play a prominent role for MSCs targeted in treatment of craniofacial defects [186].

Mazzola et al. believed that AdSCs use can have beneficial clinical applications in otolaryngological practice, such as treatment of vocal fold augmentation in glottic incompetence, and treatment of post-parotidectomy Frey syndrome and velopharyngeal insufficiency [187]. Paduano et al., in their review, reported successful use of AdSCs in craniomaxillofacial regeneration [188]. The application of DPSCs with high plasticity and multi-potential capacity to differentiate into several different tissues, when seeded on scaffolds, have been mentioned in craniomaxillofacial bone defects [189]. Human UCSCs when seeded on calcium phosphate cement (CPC) scaffold were shown to remain viable, osteodifferentiated in the injured tissue and to enhance bone regeneration [190]. Thein-Han and Xu showed when human UCSCs were seeded onto CPC with collagen, an excellent proliferation, differentiation, and synthesis of bone minerals that can enhance bone regeneration in craniofacial injuries [191]. Tang et al. reported that MSCs and iPSCs had good viability and osteogenic differentiation, when seeded onto CPC scaffold and could promote bone regeneration in craniofacial defects [192]. In recent years, AdSCs-based biomaterial scaffolds were shown to cover the needs of oral and maxillofacial tissue engineering because of their superior performance [193].

## 20. Craniofacial Clinical Trials

There are two phase I/II clinical trials registered on the official clinical trial website (www.//clinicaltrials.gov, accessed on 7 April 2021) based on the use of autologous AdSCs and manufactured bone substitute of Bonofill in regeneration of maxillofacial bone defects. Bonus BioGroup (Haifa, Israel) in a phase I/II open label single center clinical study evaluated the safety and the efficacy of autologous adipose tissue derived cells (BonoFill) as bone filler in reconstructing the bone void in the maxillofacial area of eleven 18–65 years old patients. They showed the procedure to be safe without any chronic bone infection (osteomyelitis) in absence of any significant changes in complete blood count (CBC) and general health. The bone regeneration in the operated site was significantly accelerated. The bone defects/voids were filled with a significant amount of bone tissue (NCT02153268) [194]. Bonus BioGroup (Haifa, Israel) in a phase I/II open label single center clinical study evaluated the safety and efficacy of BonoFill-II in reconstructing maxillofacial bone of twenty 18–80 years old patients. They reported no treatment-related adverse events, such as osteomyelitis or significant changes in CBC and general health. The bone regeneration in the operated area was significantly accelerated and the bone defects/voids were filled with a significant amount of bone tissue too (NCT02842619) [195]. Wildburger et al., in a split-mouth design of seven patients with bilateral highly atrophic posterior maxilla transplanted BMSCs or pure bovine bone material, showed no significant difference in new bone formation between treatments [166]. Table 8 demonstrates clinical trials in treatment of craniofacial defects and diseases based on stem cell source and year of study.

## 21. Craniofacial Case Reports

Limited case reports are available with encouraging results using AdSCs to treat defects in the calvaria [194], mandible [195] and maxilla [196]. The first clinical report in bony tissue for use of AdSCs was described by Lendeckel et al., who used stem cells together with autologous fibrin glue to augment the bone tissue and to reconstruct a large post-traumatic bone defect with a good ossification [194]. Mesimaki et al. utilized GMP-grade human autologous AdSCs in combination with tricalcium phosphate (TCP) and BMP-2 to reconstruct a large maxillary defect that expressed osteogenic-related markers, including osteocalcin, osteopontin, ColI, and RUNX-2 [196]. Eight patients (4 women and 4 men, age 29–55 years), with pronounced atrophy of the bone tissue, received AdSCs seeded on scaffold and showed cell transplantation to be a safe procedure allowing rapid organotypic recovery of the lost tissue [195]. Sandor et al., by using AdSCs seeded on bioscaffolds in combination with BMP-2 in patients with large craniofacial bone defects, showed extremely encouraging results for reconstruction of craniofacial osseous defects [197].

Another clinical report by Sandor et al. revealed that application of AdSCs could treat a large bone defect at the mandibular symphysis with successful integration of the construct to the surrounding skeleton [198]. GMP-level AdSCs in mandibular ameloblastoma resection defects of three patients resulted in reconstruction of the injured tissue [199]. Sandor et al., in another clinical study, successfully used a combination of autologous AdSCs, with four different scaffolds, and showed reconstruction of complex mandibular defects [200]. Human AdSCs when seeded on hydroxylapatite-collagen hybrid (Coll/Pro Osteon 200) (Zimmer Biomet, Warsaw, IN, USA) scaffold led to bone regrowth and skeletal development in patients with zygomatic and maxillary defects [201]. Rajan et al. using cell therapy of autologous BMSCs seeded onto b-TCP reported successful upper jaw reconstruction of a patient following maxillary injury [202]. Table 9 illustrates case reports regarding treatment of craniofacial defects and diseases based on stem cell source and year of study.

## 22. Craniofacial In Vivo Studies

Lee et al., by using implanted AdSCs with BMP-2 and bioscaffold grafts in rats with large mandibular defects, illustrated healing of critical-sized segmental mandibular defects [203]. In damaged salivary gland tissue of mice after irradiation, human AdSCs could ameliorate radiation-induced tissue damage [204]. Watanbe et al. used AdSCs with and in absence of collagen scaffold to regenerate a 7 mm gap in the rat facial nerve, while the utilized cells were differentiated into Schwann-like cells and neuroregeneration happened [205]. When rats were exposed to radiotherapy to replicate this pathology, it showed that injection of human AdSCs into submandibular salivary glands could improve salivary gland flow rate [206]. In mandibular defects of rabbits, fibrin glue, associated with AdSCs, could significantly increase the thickness of new cortical bone and accelerate the healing process [182].

A combination of AdSCs with fibrin glue scaffold in rabbit mandibular defects was illustrated to have repairing therapeutic effect, while the impact of AdSCs/fibrin glue on bone formation was greater than that of fibrin glue alone [182]. Sha et al. indicated that implanted human BMSCs with three-dimensional hydroxyapatite/poly-d/l-lactide [3D-HA/PDLLA] composite scaffold in mandibular critical defect of rats exhibited good osteoconductivity and an adequate blood supply to facilitate bone regenerative and reconstruction of maxillofacial boney defect [207]. Lee et al. demonstrated that DPSCs, seeded on Bio-Oss scaffold, in rabbit matched the bone regeneration efficacy similar to use of BMSCs indicating a promising strategy for craniofacial defect repair [208]. Tissue-specific stem cells from the human submandibular salivary gland (hSGSCs) that expressed MSC surface antigen markers in radiation-damaged rat salivary glands could rescue hyposalivation and body weight loss, restore acinar and duct cell structure, and decrease the amount of apoptotic cells; advances in humans still remain speculative [209]. Table 10 exhibits in vivo studies of craniofacial defects based on stem cell source and year of study.

## 23. Limitations by Use of MSCs in Otorhinolaryngology

There are some limitations in MSC administration in otorhinolaryngology and treatment of defects and diseases in head and neck regions, leading to differences in findings in various studies, such as differences in MSC sources, injected cell number, times cells were transplanted, intervals between injections, and route of administration, such as the process of MSCs relocation into the tissue and the survival of transplanted cells. The difference in induction of defects, type of animal model, the assessment methods, and the follow-up time can explain the variations in undertaken studies. It is crucial to standardize in vitro protocols and in vivo animal models to generate safe clinical MSCs for patients with otorhinolaryngeal pathologies. It is worthy to mention that other factors can also influence the outcome, including the difference in cell culture laboratories that may use diverse procedures for cell isolation and purification. The storage condition in different laboratories, regarding the lyophilization, cold chain, and transportation, is another variable that impact the results. Stem cells must be properly controlled and optimized to avoid unnecessary cell growth and infection. Additionally, the sample size is of great importance too. A low-quality method can also result in a different outcome. When viral vectors are used, they should be tested in detail for their safety to ensure that they do not affect the phenotype. Despite these limitations, stem cell therapies remain a tempting strategy in head and neck surgery, because they can overcome current obstacles and lead to tissue regeneration in otorhinolaryngeal pathologies.

In conclusion, the results from clinical trials, case reports, and in vivo studies are still encouraging, revealing that MSC transplantation can be potentially safe and, with anti-inflammatory and immunomodulatory properties, used in cell-based therapies for management and treatment of diseases and defects in otorhinolaryngology. It is also vital that clinicians to be knowledgeable of choices of MSC transplantation that may best fit the patients’ needs and improve their overall quality of life and satisfaction. More studies are nevertheless required to clarify the outcome of transplanted cells in larger sample sizes and apply standardized methodology and time scales to enable a better comparison for patients in otorhinolaryngology across the world. The future human trials work in this field are also necessary to be built upon previously completed in vivo works in an effort to move towards more human models.

## Figures and Tables

**Table 1:** In vivo studies in treatment of inner ear hearing loss.

Type of Stem Cell	Animal Model	Hearing Loss Model	Outcome	Reference
BMSCs and HSCs (EGFP labeled)	Mouse	Irradiated deafened by a single 950-cGy dose of total body	Integration of cells in the cochlea and differentiation to inner ear SLF without any adverse effects on +auditory function	[67]
BMSCs	Gerbil	Auditory neuropathy deafened with ouabain	Survival of MSCs within the modiolus, regeneration of damaged SGNs without any evidence of hyperacute rejection	[68]
BMSCs	Rat	Acute SNHL secondary to fibrocyte dysfunction [mitochondrial toxin]	Expression of connexin 26 and connexin 30, reactivation in gap junction between neighboring cells, and acceleration of hearing recovery	[69]
BMSCs (GFP-labeled)	Mouse	Normal cochleae	Settled cells within the cochlea, expression of cochlear gap-junction protein connexin 26, and restoration of cochlear cells	[71]
BMSCs	Guinea pig	Autoimmune deafened adult	Homing and survival capability of cells in cochlea, and transdifferentiation of MSCs to cochlea cell types	[72]
BMSCs	Guinea pig	Ouabain-induced auditory neuropathy	An increase in SGN number, and improvement of hearing function	[73]
BMSCs	Rat	Noise-induced or ototoxic SNHL	Survival of a small number of MSCs within the spiral ganglion area	[74]
BMSCs and AdSCs	Guinea pig	Deafened model	Differentiation into neuron-like cells	[75]
BMSCs	Mouse	Damaged SLF network	Functional hearing recovery after cell transplantation	[76]
BMSCs (EGFP)	Mouse	SNHL	Cell migration to cochlea and differentiation into SLF in absence of adverse effects on auditory brainstem response	[77]
BMSCs	Guinea pig	Neomycin-deafened	An increase in number of SGNs in organ of Corti and spiral ganglion and differentiation into neuronal progenitor cells and neuronal cells, treatment of SNHL	[78]
BMSCs (Magnetic labeled)	Guinea pig	Cochleostomy deafened	Successful engraftment in the inner ear	[79]
BMSCs	Mammalian	Structural reorganization of the damaged cochlea	improve incomplete hearing recovery	[80]
BMSCs	Mouse	Degeneration of cochlear fibrocytes in the spiral ligament [SL] using local application of 3-nitropropionic acid [3-NP]	Regeneration and maintenance of fibrocytes in damaged spiral ligaments, partial restoration of cochlear function	[81]
BMSCs	Rat	Cochlear insult	No recruitment of inflammatory leukocytes and edema in the cochlea	[82]
AdSCs	Guinea pig	Noise deafened adult	Cell survival and migration at the site of tissue damage, and treatment of deafness	[83]
AdSCs	Mouse	Autoimmune hearing loss model by β-tubulin	Immunomodulatory properties, absence of atrophy in stria vascularis or organ of Corti, and improvement in hearing function	[84]
AdSCs	Rat	Kanamycin deafened	Elevations of BDNF, significant number of MSCs in the cochlea, improved survival of SGNs, and improved hearing threshold levels	[85]
UCSCs	Guinea pig	SNHL deafened by neomycin and ouabain octahydrate into middle ear	A rise in the number of SGNs, improvement in hearing thresholds	[86]
TSCs	Mouse	Noise deafened	Attenuating ototoxic effects of noise trauma	[87]
oeMSCs	Mouse	Lateral wall cochleostomy hearing loss	Survival of implanted stem cells, transdifferentiation into SLF and conservation of hearing	[88]
oeMSCs	Rat	Noise-induced hearing loss	Cell migration around the SGN and restoration of hearing loss	[89]
oeMSCs	Mouse	Lateral wall cochleostomy with early onset progressive SNHL	A reduction in inflammation, oxidative stress and cell apoptosis, significant lower hearing threshold levels and amelioration of hearing loss	[90]
NSCs	Rat	Spiral ganglion loss	Neuronal differentiation, repair in injured cochlea	[91]

BMSCs: Bone marrow-derived stem cells. AdSCs: Adipose tissue-derived stem cells. TSCs: Tongue-derived stem cells. HSCs: Hematopoietic stem cells. UCSCs: Umbilical stem cells. oeMSCs: Olfactory epithelium neural stem cells. NSCs: Nasal tissue-derived stem cells. SLF: Sensory fibrocyte-like cells. SNHL: Sensorineural hearing loss. SGNs: Spiral ganglion neurons. NF-200: neurofilament-200, MAP-2: Microassociate protein-2, NeuN: Neuron-specific nuclear protein, GFAP: Gial fibrillary acidic protein, GFP: Green fluorescent protein. EGFP: Enhanced green fluorescence protein. BDNF: Brain-derived neurotrophic factor.

**Table 2:** In vivo studies of tympanic membrane related hearing loss.

Type of Stem Cell	Animal Model	Tympanic Hearing Loss Model	Outcome	Reference
BMSCs	Rat	Perforated TM	A decrease in the stiffness of the healed tympanic membrane and healing process with an enhanced restoration	[101]
BMSCs, GFP-labeled embedded in porcine GS, hyaluronate-derived ED and PVA	Mouse	Injured TM	Transplanted cells were deposited in the injured tissue, differentiated into SFL, formation of a thicker neotympanum	[102]
BMSCs seeded on HA	Mouse	Bilateral large tympanic perforations	Goncalves et al. by use of Formation of an intact neotympanum, repair and restoration of trilaminar structure in TM, and neo-tympanal integrity and transparency	[103]
BMSCs with a 3D bioprinted scaffold [polycaprolactone/collagen/alginate]	Rat	Subacute TM perforations	Recovery of the hearing capacity at all frequencies, regeneration of the thick neodrum with membrane stiffness and stability, an acceleration in TM healing process	[104]
AdSCs	Mouse	Sub-total pars tensa perforations	Secretion of growth factors, a promoted significant keratinocyte proliferation and migration and TM wound healing	[105]

BMSCs: Bone marrow-derived stem cells. AdSCs: Adipose tissue-derived stem cells. TM: Tympanic membrane. GS: Gelita-Spon. ED: EpiDisc. PVA: polyvinyl alcohol. SFL: Sensory fibrocyte-like cells. HA: hyaluronic acid. 3D: Three-dimensional.

**Table 3:** In vivo studies of laryngeal defects and disorders.

Type of Stem Cell	Animal Model	Defect Model	Outcome	Reference
DPSCs seeded on naïve nanofiber scaffold	Pig	Laryngeal defect	Formation of neocartilage tissue	[113]
BMSCs seeded on de-cellularised hemi-larynx	Pig	Full-thickness defect created in the cricoid cartilage	Vascularization and orthotopic implantation without adverse effects on respiration, swallowing or vocalization and formation of contiguous epithelium and a rudimentary vocal folds	[114]
BMSCs	Dog	Laryngotracheal stenosis	Complete epithelialization with minimal chronic inflammatory cell infiltration in submucosa of vocal folds	[115]
BMSC seeded on Porcine hemi-larynx de-cellularized	Pig	Defective thyroid cartilage	No adverse effect on respiratory function, swallowing and vocalization, and complete epithelialization of the mucosal surface and the development of rudimentary vocal folds	[107]

DPSCs: Dental pulp stem cells. BMSCs: Bone marrow-derived stem cells.

**Table 4:** Undertaken clinical trials in treatment of vocal fold scarring using MSCs.

Type of Study	No. of Patients	Stem Cell Source (*n*)	Outcome	Reference
Clinical trial phase I	Sixteen 18 years and older with severe hoarseness and vocal fold scarring	BMSCs with a hyaluronan gel	Feasibility, safety, and efficacy of the procedure and functional measures, improved healing of scarred vocal cord one year postoperatively	[123]
Clinical trial	Eight 18 years and older with vocal fold scarring and dysphonia	AdSCs	Positive therapeutic effect of cell transplantation, improved healing of scarred vocal cord	[124]
Clinical trial	12 patients aged 16–66 years with laryngeal hemiplegia or defects	AdSCs	Therapeutic efficacy of cell transplantation in restoration of glottic competence	[125]

BMSCs: Bone marrow-derived stem cells. AdSCs: Adipose tissue-derived stem cells.

**Table 5:** In vivo studies of vocal fold injuries.

Type of Stem Cell	Animal Model	Defect Model	Outcome	Reference
BMSCs with atelocollagen	Dog	Injured vocal fold	Beneficial effect in regeneration of the injured vocal fold	[126]
BMSCs	Mice (GFP transgenic)	Resected vocal folds	Cell survival in host tissue, positive expression for keratin and desmin markers of epithelial and muscular tissues, improvement in quality of healing process in vocal fold injuries	[127]
BMSCs	Rabbit	Scarred vocal folds	Improved viscoelastic parameters and less signs of scarring expressed as collagen content	[128]
BMSCs seeded on decellularized scaffolds with growth factors	Rat	Scarred vocal fold	Favorable ECM production, myofibroblast differentiation, hyaluronan metabolism, production of TGF-β1in absence of any cytotoxicity and preservation of local cell proliferation, Graft survival, and functionality and safety reconstruction of vocal folds	[129]
BMSCs	Rabbit	Scarred vocal folds	Improvement in healing of the vocal fold, reduced lamina propria thickness and collagen type I content and restoration of viscoelastic function	[130]
BMSCs	Rabbit	Vocal fold scar	Enhancing the functional healing of the vocal fold with decreased lamina propria thickness and restoration of viscoelastic shear properties	[131]
BMSCs and atelocollagen	Dog	Vocal fold scarring	An increased HA distribution and a decreased dense collagen deposition in the lamina propria, better mucosal vibration	[132]
BMSCs	Rabbit	Vocal fold injury	Cell survival in injured vocal folds, favorable and enhanced wound healing in vocal folds	[133]
BMSCs and AdSCs GFP-labeled with HA	Rat	Vocal fold scar	Equal regenerative and restoration effects of both stem cells, identical upregulation of FGF2 and Has3, AdSCs upregulated HGF more	[134]
BMSCs with porcine gel	Rabbit	Scarred vocal folds	Prolonged survival of BMSCs in vocal folds, and promoting a scarless vocal folds healing	[135]
BMSCs	Rabbit	Vocal fold scarring	Early resolution of viscoelasticity and a decrease in inflammation and facilitation of tissue repair	[136]
BMSCs	Rat	RLN transection injury	Complete recovery of vocal fold mobility and functional recovery	[137]
AdSCs together with atelocollagen	Dog	Injured vocal fold	Regeneration of injured tissue and preventive activity in vocal fold scarring and atrophy	[138]
AdSCs with HA and HGF	Rabbit	Injured vocal fold	Preventing vocal fold atrophy after laryngeal surgery	[139]
AdSCS	Pig	Unilateral vocal fold paralysis	Noticeable hypertrophy in thyroarytenoid muscle fiber, improvement in unilateral vocal fold paralysis	[140]
AdSCs with collagen or HA	Rabbit	Vocal fold wound	The facilitatory role of stem cells in vocal fold regeneration, regulating the generation and orderly distribution of ECM	[141]
AdSCs	Rabbit	Injured vocal folds	A decreased collagen content, fewer signs of scarring, an increase in viability of stem cells in vocal folds and an improvement in wound healing	[142]
AdSCs	Rabbit	Vocal fold injury	Vocal fold regeneration	[143]
AdSCs seeded on alginate-HA hydrogel	Rabbit	Vocal fold wound	Prolongation of the retention time of stem cells in the vocal folds and a promotion in wound healing	[144]
AdSCs	Dog	Acute vocal fold wound	Ability of cells to secrete ECM, vocal fold vibration recovery and improvement in vocal fold wound healing	[145]
AdSCs and fibrin hydrogel	Rabbit	Scarred vocal folds	Minor evidence of scar formation and immune reaction after transplantation, Vibration and a complete reconstruction in vocal fold cover layer	[146]
AdSCs	Rabbit	Chronic vocal fold scar	An enhanced healing in vocal folds and a reduction in scar tissue	[147]
AdSCs	Rabbit	Acute vocal fold scar	An improved vocal fold healing	[148]
AdSCs	Rabbit	Chronic vocal fold scar	Enhancing the healing of the vocal fold injury and the reduction in the scar tissue	[147]
Muscle stem cells	Rat	Vocal fold paralysis	Myoblast survival and attenuation of muscle atrophy	[149]
Muscle stem cells	Rat	Vocal fold paralysis	Enhancement of MSC survival and promotion of neural regeneration	[150]
Muscle stem cells	Rat	RLN transection injury	An enhanced reinnervation state	[151]
LSCs	Dog	Vocal fold injury	Differentiated into myofibroblasts and fibroblasts, regulation of ECM, inhibiting the rapidly decrease in elastic fiber and HA, decreasing the microenvironment inflammatory reaction, blocking collagen and the fibronectin rapid increase, and preventing the formation of vocal fold scar	[152]

AdSCs: Adipose tissue-derived stem cells. BMSCs: Bone marrow-derived stem cells. LSCs: laryngeal stem cells. FGF2: Fibroblast growth factor 2. HGF: hepatocyte growth factor. GFP: Green fluorescent protein. ECM: Extracellular matrix. HA: Hyaluronic acid. TGF-β1: Transforming growth factor beta 1. RLN: Recurrent laryngeal nerve.

**Table 6:** Undertaken clinical trials in treatment of sinuses diseases using MSCs.

Type of Study	No. of Patients	Stem Cell Source (*n*)	Outcome	Reference
Clinical trial	Seven patients with a loss of height in the posterior maxilla	BMSCs in combination with biphasic HA/TCP	A viable therapeutic alternative for implant placement	[163]
Randomized, controlled clinical trial	12 patients with atrophic maxilla and bilateral sinus floor augmentation	BMSCs seeded on bovine bone mineral	Formation of sufficient volume of new bone and a reliable placement of implants within a time frame	[164]
A randomized controlled clinical trial	7 patients with bilateral highly atrophic posterior maxilla	BMSCs or bovine bone mineral	No significant difference in new bone formation between treatments	[166]
Phase I/II randomized, controlled clinical trial	Thirty patients with maxillary sinus deficiencies	BMSCs enriched with CD90+ stem cells and CD14+ monocytes	Safe for maxillary sinus floor reconstruction and accelerating and enhancing tissue engineered bone quality	[167]
A randomized controlled clinical trial	8 patients with maxillary sinus deficiencies	BMSCs or bovine bone mineral	An increase in bone formation in sinus lift procedures when stem cells were utilized	[168]

BMSCs: Bone marrow-derived stem cells. AdSCs: Adipose tissue-derived stem cells. HA/TCP: hydroxyl apatite/β-tricalcium phosphate.

**Table 7:** In vivo studies in rhinology.

Type of Stem Cell	Animal Model	Defect Model	Outcome	Reference
BMSCs and iPSC-MSCs	Mouse	Ovalbumin-induced allergic inflammation in upper and lower airways	A decrease in inflammatory cell infiltration, a decrease in serum levels of Th2 immunoglobulins and cytokines in bronchoalveolar and/or nasal lavage fluids and protection from allergy-specific pathological changes	[169]
BMSCs	Rat	Olfactory nerve degeneration	Accelerating regeneration of olfactory mucosa	[170]
BMSCs with chondrocytes and cellulose and alginate	Mouse	Implantation subcutaneously on the back	Chondrocyte proliferation and cartilage formation	[171]
BMSCs with chondrocytes	Mouse	Implantation in immunodeficient athymic nude model	Synergistic cartilage matrix production, termination of tissue calcification and generation of a stable implantable 3D engineered cartilage graft	[172]
AdSC	Rat	Unilateral transection of the olfactory nerve and degeneration of olfactory epithelium	Restoration of the thickness and cellular composition of epithelium, differentiation into olfactory receptor neurons and endothelial cells and a promoted regeneration in olfactory epithelium	[173]
AdSCs	Mouse	Allergic rhinitis	Inhibiting eosinophilic inflammation	[174]
AdSCs	Rabbit	Nasal injury	Abundance and density of the ciliated nasal epithelial cells and enhancing of the tissue healing	[175]
GFP-labeled UCSCs	Mouse	Allergic rhinitis induced by ovalbumin	Inhibiting the expression of the cytokines IL-10 and INF-γ and preventing allergic responses	[176]
oeMSCs	Rat	Vagus nerve section	Reinnervation in the vocal folds	[177]
Tonsil-derived stem cells	Mouse	Allergic rhinitis model	A significantly decreased allergic symptoms, a reduced infiltration of eosinophils and neutrophils in the nasal mucosa, a significantly declined IL-4 mRNA expression, and inhibition of T cell activation, MAP kinase, p65, and NFAT1	[178]
TSCs	Mouse	Allergic rhinitis induced by ovalbumin	A significantly reduced allergic symptoms, a decrease in eosinophil infiltration, a decline in total serum and IgE, a reduction in the nasal and systemic Th2 cytokine profile and an inhibition in allergic inflammation	[179]

AdSCs: Adipose tissue-derived stem cells. BMSCs: Bone marrow-derived stem cells. oeMSCs: Olfactory epithelium neural stem cells. UCSCs: Umbilical cord stem cells. GFP: Green fluorescent protein. IgE: immunoglobulin E. Th: T-helper. IL: Interleukin. MAP kinase: Mitogen-activated protein kinase. NFAT1: Nuclear factor of activated T cell 1. IFN-γ: Interferon gamma.

**Table 8:** Undertaken clinical trials in treatment of craniofacial defects and diseases using MSCs.

Type of Study	No. of Patients	Stem Cell Source (*n*)	Outcome	Reference
Phase I/II clinical trial	Eleven 18–65 years old patients with maxillofacial bone defects	AdSCs or Bonofill	The procedure was safe without any chronic bone infection, absence of changes in CBC and in general health, significantly accelerated bone regeneration and the bone defects/voids were filled with a significant amount of bone tissue	[194]
Phase I/II clinical trial	Twenty 18–80 years old patients with maxillofacial bone defects	AdSCs or Bonofill	No treatment-related adverse events, such as osteomyelitis, absence of changes in CBC and in general health, significantly accelerated bone regeneration and the bone defects/voids were filled with a significant amount of bone tissue	[195]
Phase I/II clinical trial	Thirty patients with severe bone atrophy of the upper jaw	Autologous cells enriched with CD90+ stem cells and CD14+ monocytes delivered onto a β-tricalcium phosphate scaffold	Safe therapy for maxillary sinus floor reconstruction offering potential to accelerate and enhance tissue engineered bone quality in craniofacial bone defects and deficiencies	[166]

AdSCs: Adipose tissue-derived stem cells. CBC: complete blood count.

**Table 9:** Case reports regarding treatment of craniofacial defects and diseases using MSCs.

Type of Study	Stem Cell Source (*n*)	Outcome	Reference
Case report	AdSCs with fibrin glue	Good ossification and reconstruction of bone defect	[194]
Case report	AdSCs	Being a safe procedure allowing rapid organotypic recovery of the lost tissue	[195]
Case report	AdSCs in combination with TCP and BMP-2	Expression of osteogenic-related markers, including OC, OP, ColI, and RUNX-2, reconstruction of bone defect	[196]
Case report	AdSCs seeded on bioscaffolds in combination with BMP-2	Reconstruction of craniofacial osseous defects	[197]
Case report	AdSCs	Successful integration to the mandibular symphysis with bone defect	[198]
Case report	AdSCs	Reconstruction of the mandibular ameloblastoma defects	[199]
Case report	Combination of AdSCs with four different scaffolds	Reconstruction of complex mandibular defects	[200]
Case report	AdSCs seeded on Coll/Pro Osteon 200 scaffold	Bone regrowth and skeletal development in zygomatic and maxillary defects	[201]
Case report	BMSCs seeded onto b-TCP	Successful upper jaw reconstruction	[202]

BMSCs: Bone marrow-derived stem cells. AdSCs: Adipose tissue-derived stem cells. Coll/Pro Osteon 200: Hydroxylapatite-collagen hybrid 200. b-TCP: b-tricalcium phosphate. OP: Osteopontin. OC: Osteocalcin. BMP: Bone morphogenetic proteins.

**Table 10:** In vivo studies of craniofacial defects.

Type of Stem Cell	Animal Model	Defect Model	Outcome	Reference
AdSCs with BMP-2 and bioscaffold grafts	Rat	Large mandibular defects	Healing of critical-sized segmental mandibular defects	[203]
AdSCs	Mouse	Radiation-damaged salivary glands	Ameliorating radiation-induced tissue damage	[204]
AdSCs or collagen scaffold	Rat	A 7 mm gap in facial nerve	Differentiation into Schwann-like cells and neuroregeneration	[205]
AdSCs	Rat	Radiation-damaged submandibular salivary glands	Improving salivary gland flow rate	[206]
AdSCs with fibrin glue	Rabbit	Mandibular defect	Significantly increase in thickness of new cortical bone and acceleration of healing process	[182]
AdSCs with fibrin glue	Rabbit	Mandibular defect	Repairing therapeutic effect, while the impact of AdSCs/fibrin glue on bone formation was greater	[182]
BMSCs with three-dimensional 3D-HA/PDLLA	Rat	Mandibular critical defect	Good osteoconductivity, bone regenerative and reconstruction of maxillofacial defect	[207]
DPSCs seeded on bovine bone mineral	Rabbit	Craniofacial defect	Bone regeneration and repairing efficacy	[208]
SGSCs	Rat	Radiation-damaged salivary glands	Rescue of hyposalivation and body weight loss, restoring acinar and duct cell structure, and decreasing apoptotic cells	[209]

AdSCs: Adipose tissue-derived stem cells. BMSCs: Bone marrow-derived stem cells. DPSCs: Dental pulp stem cells. SGSCs: Submandibular salivary gland stem cells. BMP-2: Bone morphogenetic protein 2. 3D-HA/PDLLA: Hydroxyapatite/poly-d/l-lactide.

## Data Availability

All data used in this review article has been taken from published articles or public sites and proper reference have been provided.
